# Quantitative assessment of signal quality and usability of EEG and EMG recordings with PEDOT:PSS-coated microneedle electrodes

**DOI:** 10.3389/fnins.2025.1706501

**Published:** 2025-12-03

**Authors:** Tomoya Yamaguchi, Yuta Kurashina, Eiji Nagahara, Sota Oshima, Naotsugu Kaneko, Kimitaka Nakazawa, Hideyuki Tanaka, Hikaru Yokoyama

**Affiliations:** 1 Institute of Engineering, Tokyo University of Agriculture and Technology, Koganei, Tokyo, Japan; 2 Department of Mechanical Engineering, School of Engineering, Institute of Science, Tokyo, Japan; 3 Department of Life Sciences, Graduate School of Arts and Sciences, The University of Tokyo, Tokyo, Japan

**Keywords:** electroencephalography (EEG), electromyography (EMG), brain, muscle, biosignal

## Abstract

**Introduction:**

Bioelectrical signals are vital indicators of physiological function, psychological status, and clinical conditions. However, traditional wet electrodes using conductive gel are time-consuming, labor-intensive, and degrade over time due to gel drying. Recently, microneedle (MN) electrodes coated with poly(3,4-ethylene dioxythiophene):poly(styrenesulfonate) (PEDOT:PSS) have been developed for high-quality signal acquisition. Although low electrode–skin impedance has been demonstrated on hairless regions, their use in electroencephalography (EEG) recordings on hairy scalp areas and quantitative comparisons with conventional electrodes in both EEG and electromyography (EMG) remain limited.

**Methods:**

We fabricated two MN electrode types of different lengths—one for EMG on hairless skin and one for EEG on the hairy scalp—and compared them with conventional wet and dry electrodes. EMG was recorded during a force-matching task; EEG was assessed via somatosensory evoked potentials. We also evaluated setup/cleanup time, comfort, and pain during EEG measurement.

**Results:**

For EMG, MN electrodes achieved significantly higher signal-to-noise ratios than conventional electrodes. For EEG, they outperformed dry electrodes and matched wet electrodes in signal quality—without using conductive gel. Although additional time was required to part hair, cleanup was faster due to the absence of gel. Pain was comparable to dry electrodes.

**Discussion:**

PEDOT:PSS-coated MN electrodes provided superior EMG signal quality and high-quality EEG signals comparable to wet electrodes even without gel. These findings suggest that PEDOT:PSS-coated MN electrodes offer a compelling balance of signal quality and user convenience, making them especially advantageous for real-world and clinical applications where time efficiency, minimal discomfort, and gel-free operation are critical.

## 1 Background

Bioelectrical signals are crucial indicators of bodily function, psychological status, and clinical pathologies. Thanks to significant advancements in bioelectric acquisition technology, electrocardiography (ECG), electromyography (EMG), and electroencephalography (EEG) have become essential tools in both clinical and neuroscience fields (Grosse et al., 2002; Teplan, 2002; Anbalagan et al., 2023). These bioelectrical signals can be recorded using two approaches: invasive and non-invasive recordings. Invasive recordings, which involve inserting electrodes directly into the body near the target site, yield high-quality signals but are generally restricted to severe clinical interventions due to the invasiveness of the procedures involved (Mercier et al., 2022). In contrast, surface bioelectrical signals are now more frequently employed as a non-invasive alternative (Xu and Xiong, 2021; Marino and Mantini, 2024; Yokoyama et al., 2024). For instance, surface EEG signals, which are most widely used modality in various fields in the above-mentioned three types of bioelectrical signals, are employed to diagnose epilepsy (Supriya et al., 2021), to brain–machine interface (BMI) technologies that compensate for impaired bodily functions (Selfslagh et al., 2019), and to further neuroscientific research on human cognitive and motor functions (Yokoyama et al., 2019; Kaneko et al., 2021).

Because surface electrodes are placed on the skin to capture bioelectric signals, they essentially function as transducers, converting ionic currents from the human body into electronic currents that are then transmitted to external electronic systems made of various metals (Tallgren et al., 2005; Alcan and Ozkendir, 2021). For the signal transmission at the electrode–skin interface, the electrode–skin interface impedance (EII) is a crucial parameter. Typically, decreased EII leads to better signal quality, an increased signal-to-noise ratio, and diminished baseline drift (Lewis et al., 2024). To lower this impedance during EEG recordings with surface electrodes, a conductive gel containing electrolytes has traditionally been applied between the skin and the electrode (Tallgren et al., 2005). However, the wet electrode approach is both time-consuming and labor-intensive—requiring gel application and hair washing before or after measurements—and it also limits recording time due to gel drying (Tallgren et al., 2005). Consequently, balancing ease of use and high-quality signals remains challenging with current wet electrodes, which in turn hinders their widespread adoption for routine clinical use and in real-world applications for health monitoring.

In recent years, numerous research efforts have focused on developing dry electrodes to address this problem (Mathewson et al., 2017; Ehrhardt et al., 2024). Dry electrodes without conductive gel are better suited for long-term brain activity monitoring thanks to no gel drying problem and for daily use and real-world applications because of the no need for gel application and hair washing. Because of the high-resistance stratum corneum (SC) and insufficient contact between the skin and the metal electrode surface, dry electrodes generally have higher skin–electrode impedance compared to wet electrodes (Mathewson et al., 2017; Fu et al., 2020). Various new materials and structures have been proposed to improve the electrode–skin contact (Tian et al., 2019; Wang et al., 2020; Li et al., 2024b), but the trade-off is the large electrode volume or footprint because the electrode size is critical for high density EEG/EMG recordings, which are required for source estimation of the EEG and EMG signals (Xing et al., 2018; Yokoyama et al., 2022; Schreiner et al., 2024).

**Figure 1:**
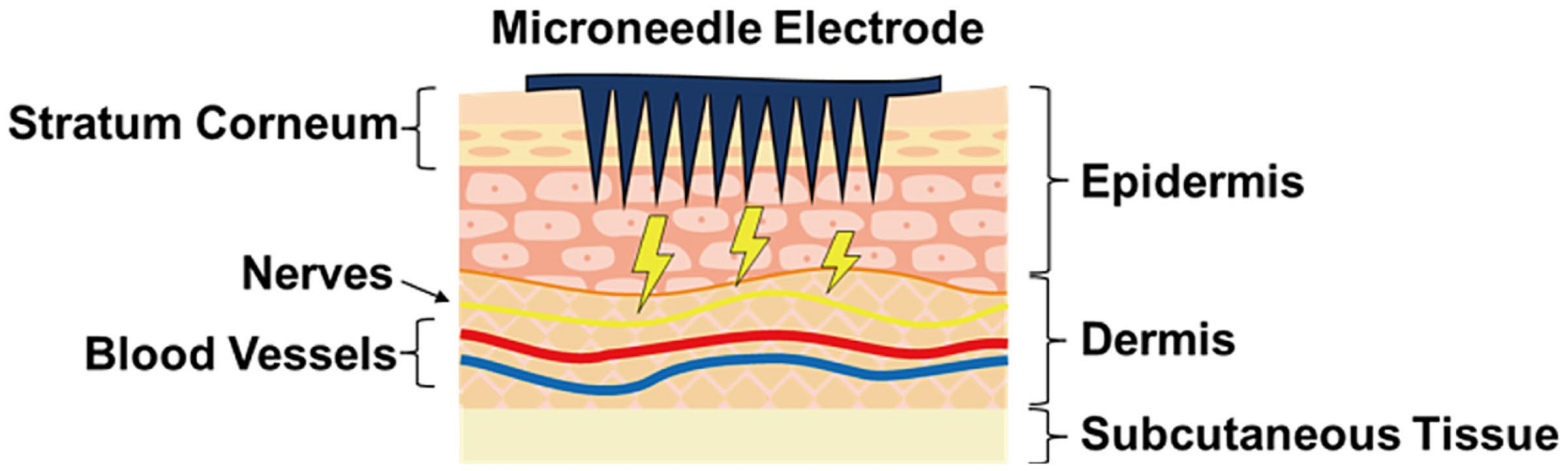
Schematic illustration of microneedle electrodes applied to human skin. The tiny needles penetrate the stratum corneum, which has high impedance, resulting in low skin-electrode impedance.

Recently developed microneedle (MN) electrodes can penetrate the high-resistance stratum corneum by using tiny needles that penetrate the skin with minimal discomfort (Wang et al., 2017; Fu et al., 2024), thus enabling high-quality measurements without the need for extensive skin preparation and conductive gel (Figure 1). Because the needles on these electrodes are very small, causing no/slight pain (Jeong et al., 2017), they are known as micro-invasive electrodes. Recently, it was reported that depositing a conductive polymer, poly (3,4-ethylene dioxythiophene):poly(styrenesulfonate) (PEDOT:PSS), onto metal MNs dramatically reduces electrode impedance (Li et al., 2022). Thus, these electrodes can be smaller than—thanks to the low impedance per size—conventional wet electrodes. PEDOT:PSS exhibits mixed conductivity, which can conducts both electronic and ionic charges, it functions as a transducer that facilitates smooth current flow between the body’s ionic environment and the electrode’s electron-conductive metal (Bianchi et al., 2022).

A study developing microneedle electrodes coated with PEDOT:PSS found that the electrode–skin impedance in hairless areas (such as the forehead or arms) was much lower compared to wet electrodes (Li et al., 2022). However, these electrodes have not yet been applied to the scalp, which is covered with hair. In addition, there has been no comprehensive quantitative comparison in EEG and EMG signals with conventional dry and wet electrodes. Although the study by Li et al., (2022) evaluated EMG signal amplitude and signal-to-noise ratio (SNR) against conventional electrodes, several methodological issues arose. For instance, electrode placement differed between electrode types—even though EMG signals vary by location (Yokoyama et al., 2021)—and no standardized muscle contraction level was used, potentially causing inconsistent muscle activity across tasks. In this study, we aimed to quantitatively compare the performance of MN electrodes coated with PEDOT:PSS against conventional wet and dry electrodes. First, to assess electrode performance under hairless conditions, we measured EMG signals and impedance on the arms. Next, we evaluated their performance in EEG signals measured from hairy regions by somatosensory evoked potentials (SEPs). During these measurements, we recorded setup and cleanup times, as well as any pain or discomfort reported by participants, to assess usability across electrode types.

## 2 Methods

### 2.1 Overview of MN electrodes

**Figure 2:**
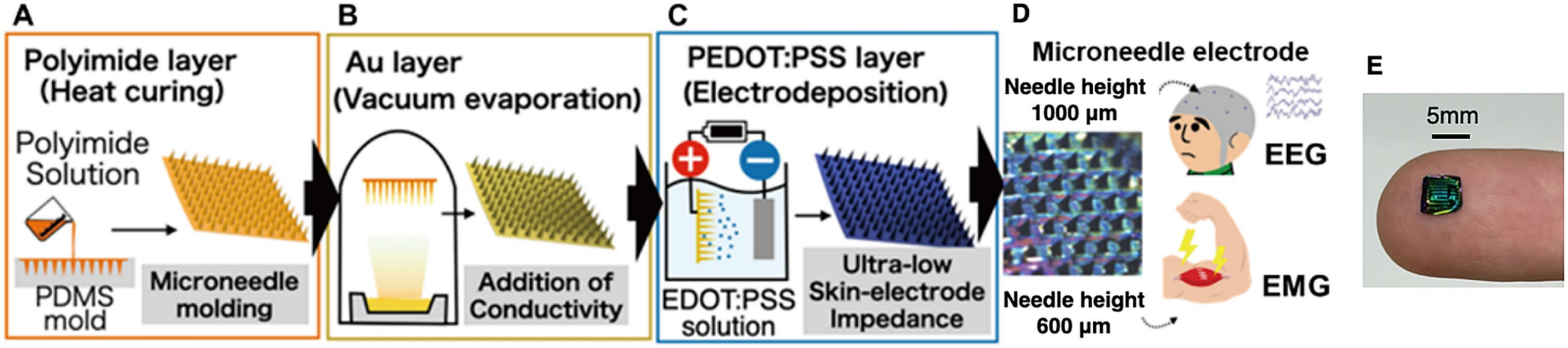
Fabrication process of PEDOT:PSS-coated microneedle electrodes for EEG and EMG recordings. **(A)** A polyimide layer formed by thermal curing of polyamic acid. **(B)** An Au layer deposited via vacuum evaporation. **(C)** A PEDOT:PSS layer formed by electrodeposition from a monomer solution. **(D)** Needle height was adjusted according to application—1,000 μm was adopted for EEG and 600 μm for EMG to account for hair thickness. **(E)** Example of a fabricated microneedle electrode; the one shown is designed for EMG recordings.

Figure 2 illustrates the flowchart of the fabrication process for PEDOT:PSS-coated MN electrodes. Based on a previous study (Li et al., 2022), we fabricated the MN electrode composed of three layers: (1) a polyimide substrate layer providing flexibility, mechanical strength; (2) a Au layer to provide electrical conductivity (Figure 2B); and a PEDOT:PSS conductive polymer layer (Figure 2C), which decrease skin-electrode contact impedance.

### 2.2 Materials

A polyamic acid solution (Pyre-M.L) was purchased from IST Corp. (Japan). The PDMS-based silicone mold for MN electrode (Mpatch Microneedle Template) was obtained from Micropoint Technologies Pte Ltd. (Singapore). Au and chromium for vapor deposition were purchased from Niraco Inc. (Japan) and PSS (molecular weight of ~ 70,000) along with EDOT (97%, the monomer for PEDOT) were purchased from Sigma-Aldrich LLC (United States).

### 2.3 Fabrication process

Two types of PDMS molds were used to fabricate MN arrays. For EMG experiments, the mold had a needle length of 600 μm, a base diameter of 200 μm, a pitch of 500 μm, and a 10 × 10 needle array. For EEG experiments, the mold had a needle length of 1,000 μm, a base diameter of 250 μm, a pitch of 500 μm, and a 15 × 15 needle array, considering hair layer thickness. The EMG-type MN array (600 μm length) was selected based on previous EMG studies (Tang et al., 2023) using commercially available PDMS molds. The EEG-type MN array employed a 1,000 μm mold purchased, chosen after preliminary testing revealed that shorter needles were insufficient for stable scalp contact through hair.

#### 2.3.1 Polyimide layer

Figure 2A illustrates the fabrication process of the polyimide layer.Each PDMS mold was ultrasonically cleaned (120 kHz with a Ultrasonic Cleaner HFC-3D, AS ONE, Japan) for 30 min and then dried. Next, polyamic acid solution was poured into the PDMS mold and centrifuged at 3,000 rpm for 15 min to ensure complete filling with a centrifugal machine (H-19a, KOKUSAN, Japan). Additional polyamic acid solution was then applied, and a scraper was used to level it to the mold’s edges. The mold was placed on a hot plate at 100 °C for 30 min to evaporate the solvent. The temperature was gradually raised to 200 °C over a 30-min period, and then held at 200 °C for another 30 min to induce imidization and cure the film. Because the PDMS mold can only tolerate up to 200 °C, the polyimide substrate was removed from the mold before an additional 5-min heating step at 300 °C, completing the imidization process and yielding the final polyimide layer.

#### 2.3.2 Au layer

Using a vacuum deposition device (VE-2013, Vacuum Device Inc., Japan), we coated both sides of the polyimide layer with a 10 nm chromium (Cr) adhesion layer and a 200 nm Au conductive layer, thereby producing conductive MN electrodes (Figure 2B). Deposition was performed separately on each side to ensure conductivity on both the front and back surfaces.

#### 2.3.3 PEDOT:PSS layer

Figure 2C illustrates the fabrication process of the PEDOT:PSS conductive polymer layer. A 2.5 wt% PSS solution and 0.01 M EDOT were dissolved in pure water, and a PEDOT:PSS layer was deposited onto the Au surface via electrochemical polymerization. In this setup, a platinum rod served as the cathode, while the MN electrode (with its gold layer) was used as the anode. Because excessive current can accelerate polymerization too quickly—leading to cracks or poor adhesion in the PEDOT:PSS layer (Li et al., 2022)—the current was increased stepwise at 20, 40, 60, and 80 μA for 150 s each, and then 100 μA was applied for 600 s. Matlab (MathWorks, United States) was used to control the current, and an NI-9265 (National Instruments, United States) functioned as the constant current source.

### 2.4 EMG experiment

#### 2.4.1 Participant

Eleven healthy adults (eight males, three females) participated in this experiment. All participants provided written informed consent for participation in the study, which was conducted in accordance with the Declaration of Helsinki and approved by the Ethics Review Committee of The University of Tokyo (reference number, 23–396).

#### 2.4.2 EMG acquisition

Because MN electrodes create small punctures in the skin, measurements were performed in the following order: wet electrode, dry electrode, and then MN electrode. Before each measurement, the skin was cleaned with an alcohol swab and dried to maintain consistent skin conditions.

EMG signals were recorded using an electromyography amplifier (BrainAmp-ExG MR, Brain Products, Germany) at a sampling rate of 2,500 Hz. The data were then processed with a fourth-order Butterworth bandpass filter (20–350 Hz), and 50 Hz powerline noise was removed with a notch filter. Two electrodes were placed on muscle belly of the biceps brachii of the dominant arm with a 2 cm center-to-center spacing, and a ground electrode was positioned on the acromion. To minimize external noise, the wires were twisted. The positions of the electrodes were marked to ensure consistent placement for each electrode condition. After attaching each electrode to the arm, it was fixed with an elastic underwrap.

All electrodes were standardized to a 5 mm × 5 mm square area. The dry electrode consisted of a round, gold-plated metal body onto which a thin insulating film with a 5 mm × 5 mm square opening was affixed; a rod on the back allowed for clip-connector wiring. To form the wet electrode, we attached a double-sided adhesive urethane foam—also featuring a 5 mm × 5 mm square opening—to the same round, gold-plated metal base used for the dry electrode, then filled the opening with conductive paste (AC cream, OT Bio Elettronica, Italy). The MN electrode was fabricated as a 5 mm × 5 mm MN array consisting of 10 × 10 needles. A silver-paste-based conductive epoxy (CW2400, Chemtronics, United States) was applied to the back of the needle surface to enable wiring.

#### 2.4.3 Experimental task

**Figure 3:**
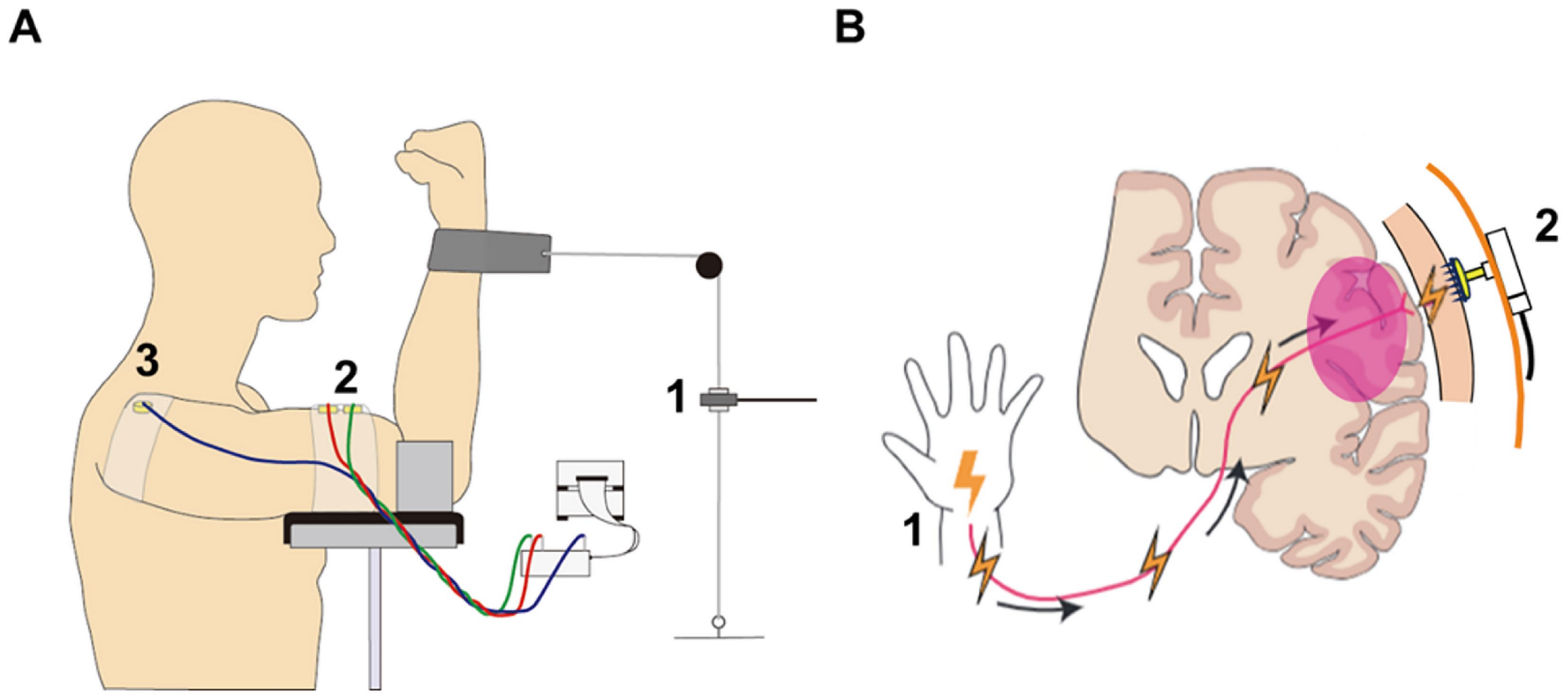
Schematic illustration of EMG and EEG experiment. **(A)** In EMG experiment, participants performed a force match task of the elbow flexion. The exerted force was measured by a force sensor (A1) and visually feedbacked to the participant. Two electrodes were placed on muscle belly of the biceps brachii (A2), and a ground electrode was positioned on the acromion (A3). **(B)** In EEG experiment, somatosensory evoked potentials were evaluated. Electrical stimulation was delivered to the right median nerve (B1) and the sensory information was propagated and reached to the somatosensory cortex. The EEG electrodes measured the sensory evoked potentials (B2).

Participants performed a force match task of flexion of the elbow joint (Figure 3A). Before the main task, maximum voluntary contraction (MVC) force was measured for 3 s. The MVC task was performed before attaching electrode. Thus, the same contraction level was used across all the electrode conditions. Then we recorded 10 s of EMG signals under relaxed (rest) conditions. Next, the participant performed elbow flexion at 20% MVC for 10 s. We selected this contraction level because 20% MVC has been frequently used in high-density EMG studies to examine motor neuron firing(Yokoyama et al., 2022; Lundsberg et al., 2024), which is a potential application of the MN electrodes. The EMG recordings of rest and the force match task were iterated for each electrode type.

#### 2.4.4 Evaluation of signals quality of EMG and skin-electrode impedance

The root mean square (RMS) of EMG amplitude was computed for each condition, and the ratio of these RMS values served as the SNR, in accordance with Equation 1:


$$\mathrm{SNR}=20\log_{10}\frac{{\mathrm{RMS}}_{\text{signal}}}{{\mathrm{RMS}}_{\text{noise}}},$$ (1)

where RMS_signal_ is the RMS value during the force match task and RMS_noise_ is the RMS value during the rest task.

Additionally, before measuring EMG signals, the EII spectra of the electrodes were measured by a two-electrode system with an Analog Discovery 3 (Digilent, United States) and its control software WaveForms (Digilent, United States).

### 2.5 EEG experiment

#### 2.5.1 Participant

Twelve healthy adults (nine males and three females) participated in this experiment. All participants provided written informed consent for participation in the study, which was conducted in accordance with the Declaration of Helsinki and approved by the Ethics Review Committee of The University of Tokyo (reference number, 23–396).

#### 2.5.2 EEG acquisition

EEG signals were recorded at a sampling rate of 5,000 Hz using an EEG amplifier (actiCHamp Plus, Brain Products, Germany). The data were then processed with a 4th-order Butterworth bandpass filter (30–150 Hz) (Rossini et al., 1981), and 50 Hz power-line noise was removed using a notch filter. Following the International 10–20 System, Cz was used as the ground electrode, Fz as the reference, and Cp3 for measuring somatosensory evoked potentials.

**Figure 4:**
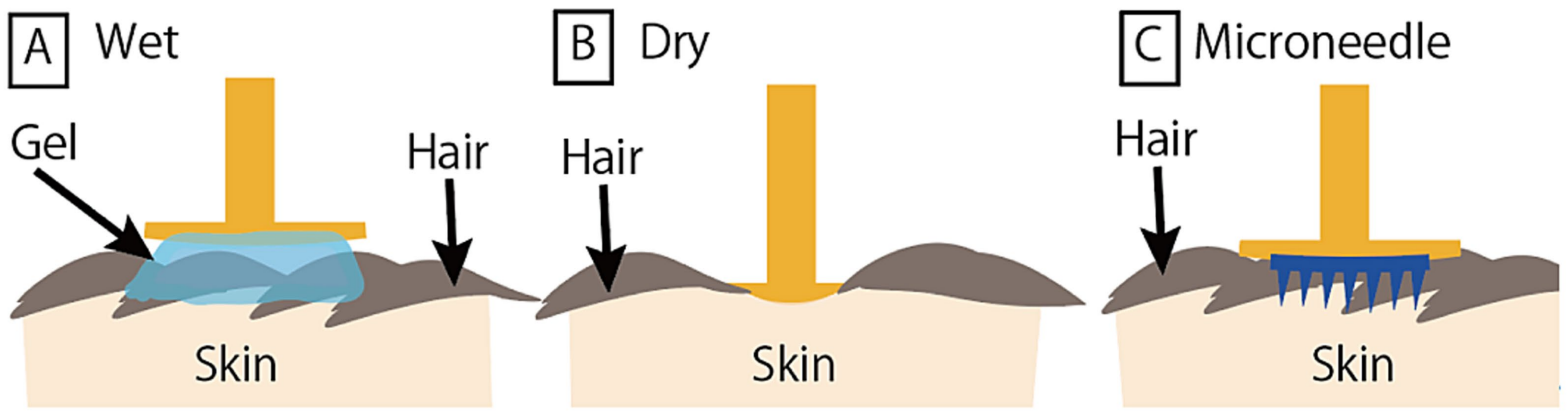
Schematic illustration of EEG recordings by different electrode types. **(A)** Wet electrode. **(B)** Dry electrode. **(C)** Microneedle electrode.

In this study, three different types of EEG electrodes (dry, wet, and MN) were compared. Measurements under these three conditions were conducted by applying gel to a dry EEG system (actiCAP Xpress Twist system) used in neuroscience research (Doerrfuss et al., 2020; Diaz-Piedra et al., 2022) to create the wet condition, and by attaching microneedle electrodes to the tip of the Dry electrode to create the microneedle condition. The details are as follows:

(1) Wet electrode (Figure 4A).

A slightly convex, dish-shaped, gold-plated metal electrode (actiCAP Xpress Twist with Flat QuickBit, Brain Products, Germany). Conductive gel (SuperVisc, Easycap GmbH, Germany) was applied between the metal surface and the skin.

(2) Dry electrode (Figure 4B).

The same actiCAP Xpress Twist system, but equipped with Long QuickBit tips (length: 12 mm) designed for hairy areas. Used without conductive gel.

(3) MN electrode (Figure 4C).

The same actiCAP Xpress Twist system with Flat QuickBit, as in the wet electrode condition. A MN array was affixed to the electrode using conductive double-sided tape (T-9426, ESD EMI Engineering, Japan), and no conductive gel was applied.

In the above comparison of electrodes during EMG measurements, the area of skin contacted by the electrodes could be controlled. However, it should be noted that in EEG measurements, such control is difficult due to the influence of hair.

Since MN electrodes can create small punctures in the skin, measurements were performed in this order: wet electrode, dry electrode, then MN electrode. Before applying the wet electrode, the skin at the electrode site was cleaned with an alcohol swab. After wet electrode measurements, the conductive gel was washed off, and the scalp and hair were thoroughly dried with a hairdryer before proceeding to the dry electrode measurement. All electrodes were then secured to an elastic cap specifically designed for actiCAP Xpress Twist.

#### 2.5.3 Electrical stimulation for somatosensory evoked potential

We administered 200 ms monophasic square-wave constant-current pulses using a clinical stimulator (USE-100, UNIQUE MEDICAL, Japan) to stimulate the right median nerve. We placed a pair of electrodes in positions on the right wrist where the visible muscle twitch was most clearly observed. The current intensity was set to evoke a 1–2 cm thumb movement, following the recommendations of the International Federation of Clinical Neurophysiology (Nuwer et al., 1994). Stimulation was applied 300 times at a repetition rate of 2.1 Hz. To maintain the participants’ attention on the stimulus, pauses were introduced every 30–50 s. Specifically, between three and four of these pauses—each lasting about 4 s—were inserted at random during each session, and participants were instructed to count the pauses and report their totals at the end of the session. All participants accurately reported the number of pauses in every session. To avoid variations in posture or hand position that could affect the experimental results, each participant’s hand and arm placements were marked on the support platform, ensuring a consistent posture across all conditions.

#### 2.5.4 Evaluation of electrode quality for EEG

To assess the quality of EEG signals recorded by MN electrodes compared to conventional (dry and wet) electrodes, we calculated the SNR of somatosensory evoked potentials (SEPs). The SEPs were obtained using a stimulation triggered averaging method, in which signal segments around the timing of electrical stimulation were extracted and averaged. The amplitude of the evoked response was defined as the N20 peak (observed between 19.0 and 21.0 ms following stimulation), and noise was defined as the standard deviation of the pre-stimulation signal from −70 to −5 ms. The ratio of these two values was taken as the SNR, in accordance with Equation 2:


$$\mathrm{SNR}=20\log_{10}\frac{{\text{Amplitude}}_{\text{signal}}}{{\mathrm{SD}}_{\text{background}}},$$ (2)

where Amplitude_signal_ is a peak amplitude of N20 and SD_background_ is the standard deviation of the pre-stimulation period (from −70 to −5 ms).

Additionally, before measuring EEG signals, the EII at 10 Hz of the electrodes were measured with an impedance measurement function of the EEG amplifier (actiCHamp Plus) and its control software (Brain Products, Germany). The analyzed frequency was predetermined by the EEG system. Since 10 Hz corresponds to the alpha wave in EEGs, impedance at 10 Hz has been used to evaluate the biopotential electrodes (Hsieh et al., 2022; Li et al., 2024a). Due to equipment limitations, any value exceeding 500 kΩ was recorded as 500 kΩ. For many participants in the dry electrode, the impedance did not fall below 500 kΩ.

#### 2.5.5 Assessment of MN electrode usability

We evaluated (1) preparation and cleanup time, (2) participant comfort, and (3) pain intensity during EEG measurements.

Preparation time was measured from the moment the experimenter began fitting the participant with an EEG cap until the electrode–skin impedance fell below 20 kΩ for the wet and MN electrodes, and below 400 kΩ for the dry electrode. In all experiments, the same experimenter (TY) was responsible for both the preparation and cleanup. If impedance for the dry electrode remained above 400 kΩ and did not improve even after parting the hair under the electrode, we stopped recording the preparation time. Cleanup time included removing the electrodes after each measurement. For wet electrodes, it also included the time needed to wash out the conductive gel and thoroughly dry the hair.

Comfort and pain were rated for each electrode condition during setup, recording, and removal using a visual analogue scale (VAS). For comfort, the highest value (10) indicated very uncomfortable and the lowest value (0) very comfortable. For pain, the highest value (10) represented the worst pain imaginable and the lowest (0) no pain at all. Participants were asked to consider overall duration, pain, and any discomfort when rating the comfort.

### 2.6 Penetration and mechanical deformation tests

Penetration tests were conducted using Parafilm® M (a flexible thermoplastic sheet, 127 μm thick, composed of an olefin-type material; Brand GMBH, Wertheim, Germany) as a skin simulant, following methods established in previous MN studies (Larrañeta et al., 2014; Alrimawi et al., 2024). Briefly, Parafilm® M was cut into a square sheet measuring 25 mm × 25 mm and stacked to form a 10-layer film with a total thickness of approximately 1.3 mm (127 μm per layer). The polyimide MN patches were automatically pressed into the film using an electromechanical universal testing machine (AG-50kNXDplus, Shimadzu Corp., Japan) equipped with a 200 N load cell (LUR-A-200NSA, Kyowa Electronic Instruments Co., Ltd., Japan) at a loading force of up to 20 N and a crosshead speed of 0.02 mm/s. The applied insertion force was determined based on a previous MN insertion study, which reported that the typical manual force exerted by a human thumb when pressing an elevator button is approximately 20 N (Larrañeta et al., 2014). After removal, the insertion performance of the MN patches was evaluated by counting the number of holes formed in each layer.

Additionally, following the procedure described by Li et al. (2022), the mechanical durability and safety of the polyimide MN patches were assessed through repeated applications on human skin. The MN patches were applied ten times to the skin of a 25-year-old male volunteer. The EMG-type MN patches were repeatedly applied to the upper arm, whereas the EEG-type patches were applied to the scalp at slightly shifted positions for each application. Changes in needle morphology before use and after one, five, and ten applications were visually examined under an optical microscope (FZ500CS, Shodensha, Osaka, Japan) following a previous study (Li et al., 2022).

### 2.7 Statistics

We compared the frequency characteristics of the impedance among the electrode types on the hairless skin during the EMG experiment. We first performed a logarithmic transformation considering homoscedasticity. One-dimensional statistical parametric mapping (SPM1d) was used to determine whether impedance differed among the electrodes (Pataky et al., 2016). The SPM analysis can find statistically significant frequency portions regarding the impedance difference among the electrodes rather than compare discrete frequency. For multiple comparisons across the three electrodes, *p*-values were adjusted using the Bonferroni method.

We also examined differences among the electrode types for the following parameters: (1) SNR during the EMG experiment, (2) SNR during the EEG experiment, (3) impedance at 10 Hz during the EEG experiment, (4) setup time, (5) removal time, (6) total time (setup + removal), (7) comfort ratings during setup, recording, and removal, and (8) pain level. Normality was tested using the Shapiro–Wilk test. The test indicated that normality was not rejected in all parameters except SNR of EMG, impedance during the EEG experiment, and pain level. Therefore, we performed a one-way analysis of variance (ANOVA) with a parametric approach to evaluate the main effect of electrode type for normally distributed data. Post-hoc comparisons were conducted using Student’s *t*-test if equal variances were confirmed, or Welch’s *t*-test otherwise. For the parameters, which did not follow the normality, we employed the Fisher–Pitman permutation test of matched pairs with 1,000 permutations for comparisons between electrode types (Boik, 1987; Yokoyama et al., 2019). All *p*-values were corrected for multiple comparisons using the Bonferroni correction.

## 3 Results

### 3.1 EMG experiment

Eleven healthy participants performed an elbow flexion force-matching task (Figure 3A). Before measuring EMG signals, the EII spectra of the electrodes was measured. Then, prior to the main task, maximum voluntary contraction (MVC) force was measured. We then recorded 10 s of EMG signals under both relaxed (rest) conditions and during elbow flexion at 20% MVC.

**Figure 5:**
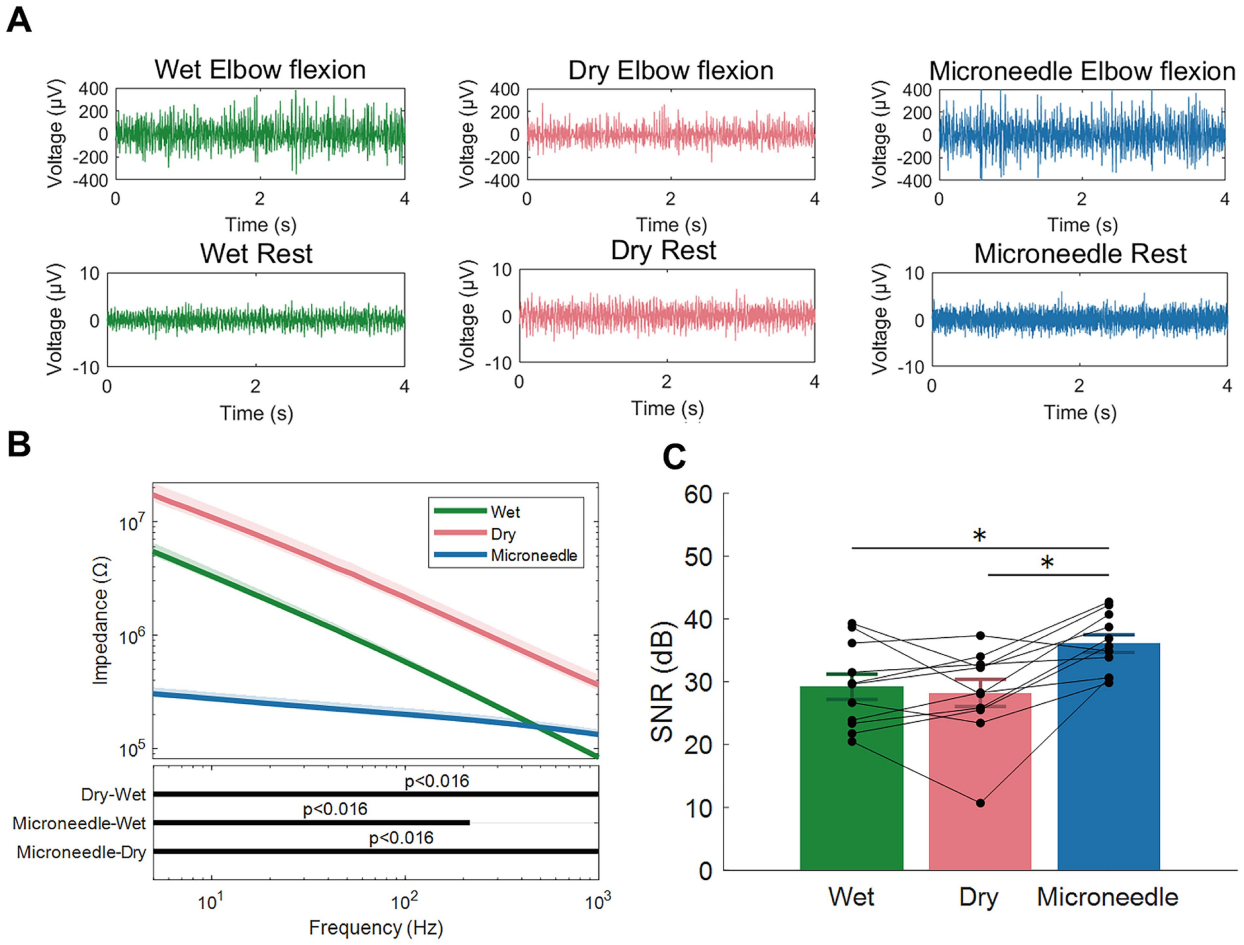
Results of three types of electrodes in the EMG experiment. **(A)** Representative examples of recorded EMG signals during elbow flexion (top row) and at rest (bottom row). **(B)** Electrode-skin interface impedance spectra for different electrodes across a frequency range from 5 Hz to 1,000 Hz (mean ± standard error). The black horizontal bars indicate frequency bands where impedance differences were significant. Transparent areas represent the standard error. **(C)** Signal-to-noise ratio (SNR) of the EMG signals. Error bars and asterisks denote the standard error and significant differences, respectively. *p < 0.05.

Figure 5A shows an example of EMG signals recorded during elbow flexion at 20% MVC (top row) and at rest (bottom row) from a participant. Figure 5B demonstrates the averaged impedance across participants. The MN electrodes had statistically lower impedance than the dry electrodes below 750 Hz, and lower than the wet electrodes below about 150 Hz (*p* < 0.05). Figure 5C showed the signal-to-noise ratio (SNR) of EMG—calculated as the EMG amplitude during elbow flexion relative to that during res. It was highest on average with the MN electrodes, followed by the wet electrodes and then the dry electrodes. Statistical analysis showed that the SNR with the MN electrodes was significantly higher (p < 0.05) than that observed with both the wet and dry electrodes.

### 3.2 EEG experiment

Twelve healthy adults participated in this experiment. Before measuring EEG signals, the EII at 10 Hz were measured with an impedance measurement function of the EEG amplifier (actiCHamp Plus, Brain Products, Germany). The analyzed frequency was predetermined by the EEG system. Since 10 Hz corresponds to the alpha wave in EEGs, impedance at 10 Hz has been used to evaluate the biopotential electrodes (Hsieh et al., 2022; Li et al., 2024a). We applied 200 ms monophasic constant-current pulses to the right median nerve using an electrical stimulator (USE-100, UNIQUE MEDICAL, Japan). Stimulation (300 trials at 2.1 Hz) was delivered via wrist electrodes at an intensity sufficient to evoke a clear thumb twitch (~1–2 cm).

**Figure 6:**
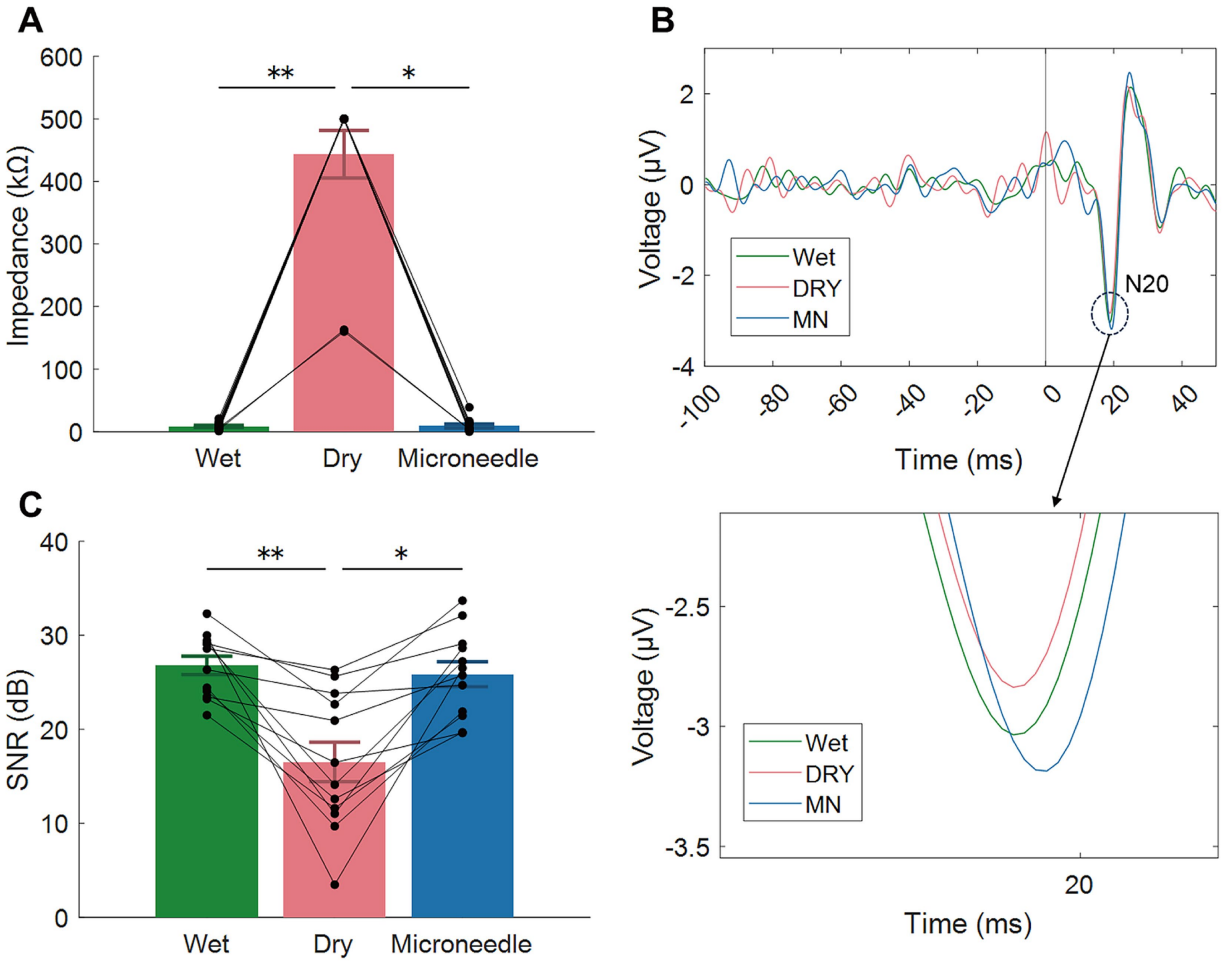
Results from three types of electrodes in the EEG experiment. **(A)** Electrode-skin impedance at 10 Hz. **(B)** Typical examples of somatosensory evoked potentials (SEP); N20 responses are highlighted by a dashed-line circle. **(C)** Signal-to-noise ratio (SNR) of the EEG signals based on the SEP responses. Error bars and asterisks denote the standard error and significant differences, respectively. *p < 0.05; **p < 0.01. Each black plot represents data from a participant.

As shown in Figure 6A, the impedance at 10 Hz was significantly lower in the wet (8.7 ± 5.6 kΩ) and MN electrodes (9.2 ± 10.3 kΩ) compared to the dry electrode (443.6 ± 126.2 kΩ, *p* < 0.05). It should be noted that, in 2 of the 12 participants, the impedance of the dry electrode exceeded the EEG system’s 500 kΩ measurement limit, even after parting the hair. There was no significant difference between the wet and MN electrodes.

Figure 6B demonstrates typical example SEP response from a participant. All the electrode showed clear response around 20 ms after the stimulation, the N20 peak was larger in the MN electrode compared to the other two electrodes and the noise level (activity during the pre-stimulus period) seemed lower in the wet electrode compared to the other two electrodes. To evaluate quality of the EEG signals among the electrodes, we evaluated the SNR of SEP (Figure 6C). The SNR was higher in the wet and MN electrodes compared to that in the dry electrode (*p* < 0.05). It did not significantly differ between the wet and MN electrodes.

### 3.3 Usability

**Figure 7:**
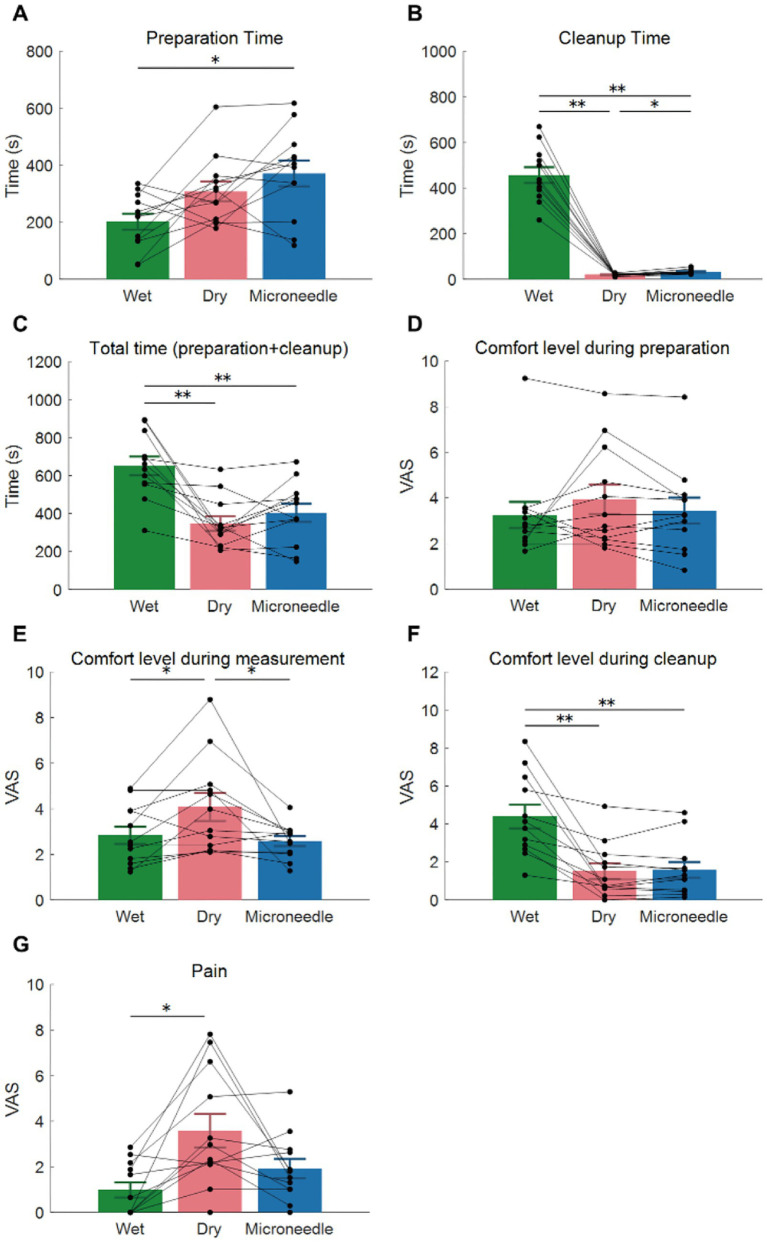
Usability scores for three types of electrodes in EEG measurements. **(A)** Preparation time until the electrode–skin impedance dropped below the target impedance (20 kΩ for wet and microneedle electrodes, 400 kΩ for dry electrodes). **(B)** Cleanup time, which included washing the hair only in the wet electrode. **(C)** Total time (preparation + cleanup). **(D–F)** Visual analog scales (VAS) assessing comfort levels during preparation **(D)**, measurement **(E)**, and cleanup **(F)**. On the VAS, 0 represents “most comfortable,” 5 “neutral,” and 10 “most uncomfortable.” **(G)** VAS for pain. 0 represents “no pain,” 10 “the worst pain imaginable.” Error bars and asterisks indicate the standard error and significant differences, respectively. Each black plot represents data from a participant. * *p* < 0.05, ** *p* < 0.01.

Preparation time was measured from the moment the experimenter began fitting the participant with an EEG cap until the electrode–skin impedance fell below 20 kΩ for the wet and MN electrodes, and below 400 kΩ for the dry electrode. In all experiments, the same experimenter (TY) was responsible for both the preparation and cleanup. Cleanup time included removing the electrodes after each measurement. For wet electrodes, it also included the time needed to wash out the conductive gel and thoroughly dry the hair. We found that the wet electrode had a significantly shorter preparation time than the other electrode types (*p* < 0.05, Figure 7A). However, it required the longest cleanup time (*p* < 0.05) compared with the other electrodes. Consequently, total time (preparation + cleanup) was significantly longer for the wet electrode than for the other two electrode types (*p* < 0.05, Figure 7C).

Regarding comfort, participants reported mild comfort levels (approximately 2 to 4 on the visual analog scale (VAS), where 0 is “most comfortable,” 5 is “neutral,” and 10 is “most uncomfortable”) across all three electrode types with no significant differences during the preparation (Figure 7D). During the measurement, participants reported feeling significantly more uncomfortable with the dry (4.1 ± 2.0) compared to the MN (2.6 ± 0.7, *p* < 0.05) and wet electrodes (2.5 ± 1.2, *p* < 0.05). In contrast, during cleanup (Figure 7F), participants indicated greater comfort with the dry [1.5 ± 1.4 (mean ± SD)] and MN (1.6 ± 1.4) electrodes compared to the wet electrode (4.4 ± 2.0, *p* < 0.05).

For pain (Figure 7G), participants reported significantly less pain with the wet electrode (0.98 ± 1.1) compared to the dry (3.6 ± 2.5, *p* < 0.05) on the VAS scale (0 = no pain, 10 = worst imaginable pain). There was no significant difference between the MN (1.9 ± 1.4) and the other two types.

### 3.4 Penetration and mechanical deformation tests

The penetration test showed that all needles of our MN electrodes for both the EEG and EMG types successfully penetrated the first layer of the Parafilm® M model (layer thickness: 127 μm). For the EMG-type MNs, which contain fewer needles (100 needles) than the EEG-type (225 needles), most needles reached the second layer (91%), and some (21%) even penetrated the third layer (Supplementary Figures 1, 2). In the EEG-type MNs, 74.2% of the needles penetrated the second layer (Supplementary Figures 1, 3). This difference can be attributed to the distribution of pressure as the number of needles increases. Since the insertion depth in the Parafilm® M model corresponds well to that in neonatal pig skin (Larrañeta et al., 2014), which is considered a reliable model for human skin (Ranamukhaarachchi et al., 2016), these results indicate that our MNs possess sufficient penetration capability for human skin.

Next, the morphological deformation of the MNs after insertion was examined. As shown in Supplementary Figures 4, 5, the morphology of the MNs showed almost no change after one use. Even after repeated applications, the needle tips showed only slight blunting without any fractures. This result is consistent with previous findings for similar polyimide MNs (Li et al., 2022). In practical use, our MN electrodes are designed for single use, thereby minimizing potential safety risks.

## 4 Discussion

We quantitatively assessed the signal quality and comfort level of newly developed PEDOT:PSS-coated MN electrodes, comparing them with conventional wet and dry electrodes. In EMG recordings, the SNR was significantly highest for the MN electrode (mean ± SD: 18.4 ± 3.2 dB), followed by the wet electrode (15.6 ± 2.8 dB, *p* < 0.05) and the dry electrode (12.3 ± 2.5 dB, *p* < 0.05). In EEG recordings, both MN (9.2 ± 2.1 dB) and wet (9.8 ± 2.3 dB) electrodes showed significantly higher SNRs than the dry electrode (5.6 ± 1.7 dB, p < 0.05). Our findings indicate that the MN electrode can deliver superior signal quality in hairless regions—surpassing even the wet electrode—and comparable performance in hairy areas. The remarkable aspect is that, despite the MN electrode being able to measure high-quality signals equal to or better than those of wet electrodes, it is a dry electrode and does not require gel. This balance of signal quality and ease of use makes the MN electrode a promising technology for both EEG and EMG applications.

### 4.1 MN electrodes on hairless region: EMG recording

In the EMG experiment, the MN electrode showed significantly lower impedance below approximately 150 Hz compared with conventional electrodes (Figure 5B), as well as a significantly higher SNR than either type of conventional electrode (Figure 5C). This suggests that MN electrodes, despite being dry-type, can record high-quality signals when used on hairless skin. Two key factors would contribute to this high signal quality: (1) the penetration of the high-resistance stratum corneum by tiny needles and (2) the mixed conductivity of PEDOT:PSS.

Regarding the first factor, MNs can indeed reduce impedance; however, MNs made solely of traditional metallic coating show higher impedance than wet electrodes (O’Mahony et al., 2016). In this study, following Li et al. (2022), we coated our MNs with PEDOT:PSS on Au, which led to a low impedance in the low-frequency band, even below that of wet electrodes (Figure 5B). This improvement occurs because the ionic/electronic mixed conductivity of PEDOT:PSS acts as a transducer, facilitating smooth current flow between the body’s ionic environment and the electrode’s electron-conductive metal (Bianchi et al., 2022).

While Li et al., (2022) did measure EMG signals using PEDOT:PSS-coated MN electrodes, their study did not standardize the measurement site and contraction force, resulting in a less robust quantitative comparison. In contrast, by controlling for the measurement site and contraction force on EMG signals, our results indicate that MN electrodes yield superior signal quality compared with widely used wet and dry electrodes (Figure 5C). Therefore, under ideal conditions—namely, in hairless regions—the MN electrode, despite being a dry type, can surpass even conventional gel-assisted electrodes in terms of signal quality.

In the comparison between conventional dry and wet electrodes, a significant difference in impedance was observed (Figure 5B). However, there was no significant difference in the SNR of the recorded EMG signals between them. We assume that this is because the difference in impedance magnitude between the dry and wet electrodes was smaller than that between the microneedle electrode and the other electrode types (Figure 5B). Consequently, a significant difference in SNR was observed between the microneedle electrode and the other electrodes, whereas no significant difference was likely to be observed between the dry and wet electrodes (Figure 5C).

### 4.2 MN electrodes on hairy region: EEG recording

Although a previous study on PEDOT:PSS-coated MN electrodes evaluated EEG signals in hairless areas such as the forehead, their usefulness in hairy regions—the most common targets of EEG measurements—remains largely unexplored. In this study, we attempted to measure EEG over a hairy area and found that although the MN electrode achieved better impedance and SNR than the dry electrode, it was comparable to the wet electrode (Figures 6B,C). This is likely because hair interferes with the electrode–skin contact, thereby reducing MN electrode performance.

When using dry-type EEG electrodes, measuring in hairy areas has long been recognized as challenging (Lopez-Gordo et al., 2014). Moreover, research on MN electrodes has shown that greater hair coverage caused higher impedance (Kawana et al., 2020). To address this, Kawana et al., (2020) developed a small shutter mechanism to separate the hair and ensure reliable electrode–scalp contact. Employing such an approach may enable PEDOT:PSS-coated MN electrodes to realize their full potential for EEG measurements in hairy regions.

Although the performance of MN electrode was similar to the wet electrode, it is noteworthy that the wet electrode used in this study was a high-grade, commercially available research system. Achieving comparable performance with a dry-type MN electrode is therefore highly significant in terms of balancing signal quality and convenience. Moreover, because MN electrodes do not require gel, they offer greater feasibility for long-term stable measurements (Wang et al., 2017). Indeed, a study using Au-based MN electrodes for EEG showed less impedance drift over extended recordings compared to wet electrodes, leading to more stable signals and improved accuracy in decoding movement intention from brain activity (Mahmood et al., 2021). Hence, even if PEDOT:PSS-coated MN electrodes do not exceed the signal quality of wet electrodes, their potential for prolonged, stable recording makes them promising for applications such as sleep research or brain–machine interface (BMI) studies, where long wearing times are common.

### 4.3 Usability

The MN and dry electrodes had a shorter cleanup time than the wet electrode (Figure 7B), because they do not require a conductive gel, allowing participants to go home immediately after removing the electrodes. On the other hand, preparation time of the MN electrodes exceeded that of the wet electrode (Figure 7A), as positioning the MN electrode took longer due to parting the hair. Incorporating a hair-separating mechanism (Kawana et al., 2020) (as mentioned previously) might automate this step and reduce setup time.

In terms of pain, although the MN electrodes induced only mild pain (1.9 ± 1.4 on the VAS scale, Figure 7G), this was higher than that was reported for the wet electrode (1.0 ± 1.1). However, the difference was not statistically significant In contrast, the dry electrodes induced significantly higher pain levels compared to the wet electrodes (3.58 ± 2.5). Because the pain level associated with the MN electrodes remained relatively low, there was no significant difference between the MN and wet electrodes in terms of comfort or discomfort during measurements, whereas participants reported feeling more discomfort with the dry electrodes (Figure 7E). Overall, despite having tiny needles, the MN electrode does not cause severe pain, and the user experience is similar to, or even better than, that of a conventional dry electrode.

### 4.4 Methodological consideration

There is a thing to note regarding our impedance results. In the hairless region, we used a frame to standardize the contact area for all electrode types, but in the hairy region, no frame was used. Consequently, gel could have spread under the electrode in the wet electrode condition, potentially creating a larger contact area than that of the MN electrode and, in turn, overestimating the wet electrode’s impedance performance.

Since the MN electrode penetrates the skin, we fixed the measurement order to wet, dry, and then MN, which may introduce order effects. In the EMG experiment, there was a concern on muscle fatigue, but previous research suggests that at 20% MVC, the biceps brachii typically becomes fatigued after about 10 min of sustained contraction (Hagberg, 1981). Because our contractions lasted only 10 s, the impact of fatigue was likely minimal. In the EEG experiment, we repeated SEP sessions in a fixed order, as in the EMG experiment. To check order effects on SEP response size, we conducted a supplemental experiment of three repeated SEP sessions using wet electrodes for all sessions. The session interval was 8 min, which is similar to the total time spent on cleanup and preparation in the main experiment (Figure 7C). The supplemental experiment showed no clear order effects on SEP response (Supplementary Figure 6).

The present study focused on feasibility and cross-sectional comparison among electrode types; therefore, test–retest reliability was not systematically evaluated. Future studies should include repeated measurements to assess intra-individual stability of electrode impedance and signal quality across sessions, which are critical for assessing reliability (Zuo et al., 2019).

### 4.5 Future direction

The MN electrodes have a potential to provide high-quality yet convenient EEG measurements, they may be very useful for clinical applications. For instance, in a previous study (King et al., 2015), walking intentions extracted from the EEG of paraplegic patients triggered electrical stimulation of leg muscles, achieving partial restoration of walking function. However, the authors noted that using a conventional gel-based wet EEG system in everyday settings was challenging, mainly due to time and effort constraints, which could limit its widespread adoption in gait rehabilitation. Although a faster setup in hairy regions remains needed, MN electrodes could simplify EEG recording while maintaining high signal quality, thereby advancing routine clinical use.

In EMG measurements, MN electrodes showed better signal quality than conventional electrodes (Figure 4C). Additionally, their low impedance suggests that reducing electrode size might still yield good-quality signals (Figure 4B). Consequently, MN electrodes could be highly valuable for high-density surface electromyography (HDsEMG), which has been actively studied recently (Del Vecchio et al., 2020; Yokoyama et al., 2022). Achieving high density EMG recordings requires miniaturizing electrodes, and the MN electrodes are well suited to that purpose. HDsEMG gains attention when used with blind source estimation, since it enables non-invasive investigation of spinal motor neuron firing, and the number of neurons that can be identified increases with higher SNR (Dai and Hu, 2019; Yokoyama et al., 2021). Therefore, applying MN electrodes to HDsEMG could increase the ability to evaluate motoneuron activity via surface EMG.

While the present study demonstrated the feasibility and quantitative advantages of PEDOT:PSS-coated microneedle electrodes, the relatively small sample size warrants cautious interpretation. Future work with larger, independent cohorts is necessary to ensure reproducibility and generalizability, in line with recent calls for enhancing reliability in neuroscience research (Marek et al., 2022).

Beyond the technical improvements, high quality bioelectric signal recordings obtained by PEDOT:PSS-coated microneedle electrodes may facilitate future investigations into spontaneous brain activity, such as the ‘dark energy’ or intrinsic slow oscillations that reflect the brain’s baseline metabolic state (Gong and Zuo, 2025). Reliable long-term EEG monitoring without gel preparation can provide a practical tool for exploring these neurobiological processes under naturalistic conditions.

## 5 Conclusion

We fabricated PEDOT:PSS coated MN electrodes and demonstrated experimentally that they can measure EMG signals with higher quality than conventional electrodes, and in EEG recordings they can match the signal quality of wet electrodes while offering dry-electrode convenience. These findings highlight the practicality of MN electrodes and indicate their strong potential for use in clinical EMG and EEG applications. It is expected that future studies apply the MN electrodes in rehabilitation BMI systems and high-density EMG recording, expanding their utility across a wide range of bio-signal measurement contexts.

## Data Availability

The raw data supporting the conclusions of this article will be made available by the authors, without undue reservation.
