# Supplementary material for: Quantitative assessment of signal quality and usability of EEG and EMG recordings with PEDOT:PSS-coated microneedle electrodes
Source: Front Neurosci. 2025 Dec 3;19:1706501. doi: 10.3389/fnins.2025.1706501 (PMC12708545; doi:10.3389/fnins.2025.1706501)
Supplement: Supplementary file 1 [file Data_Sheet_1.DOCX]

**Supplementary figures**


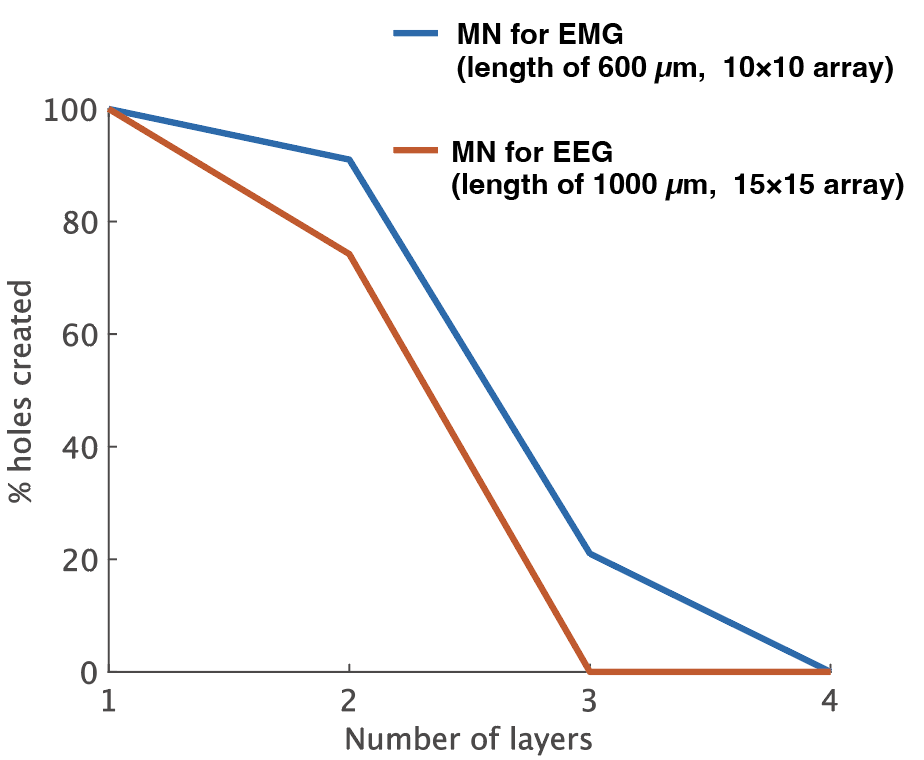


Supplementary Figure 1. Percentage of holes created in each Parafilm® M layer with insertion force at 20N for different types of MN arrays.


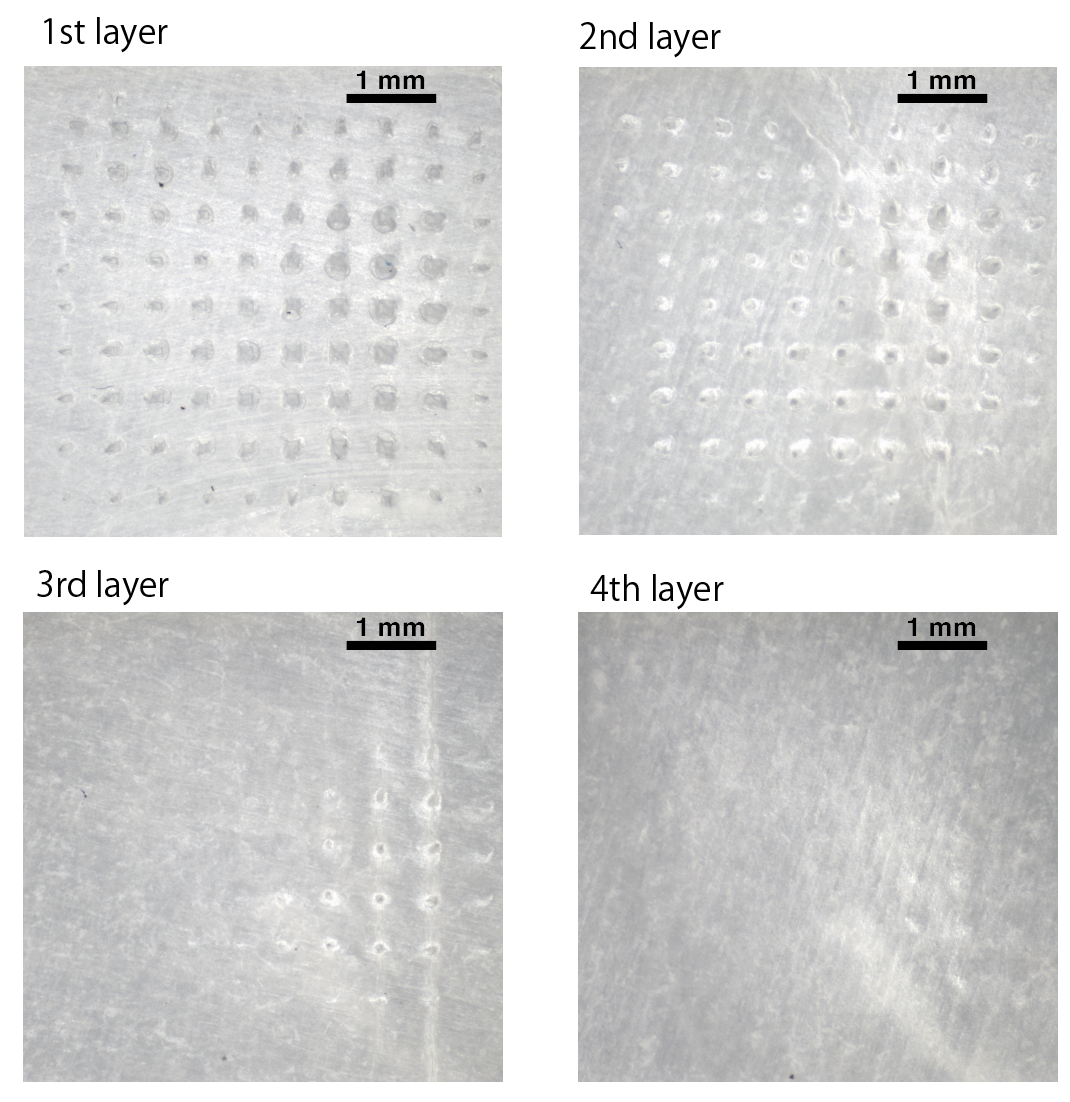


**Supplementary Figure 2.** Photograph of Parafilm® M layers after the insertion by the MN array of EMG type (600 μm, 10 × 10 needles).


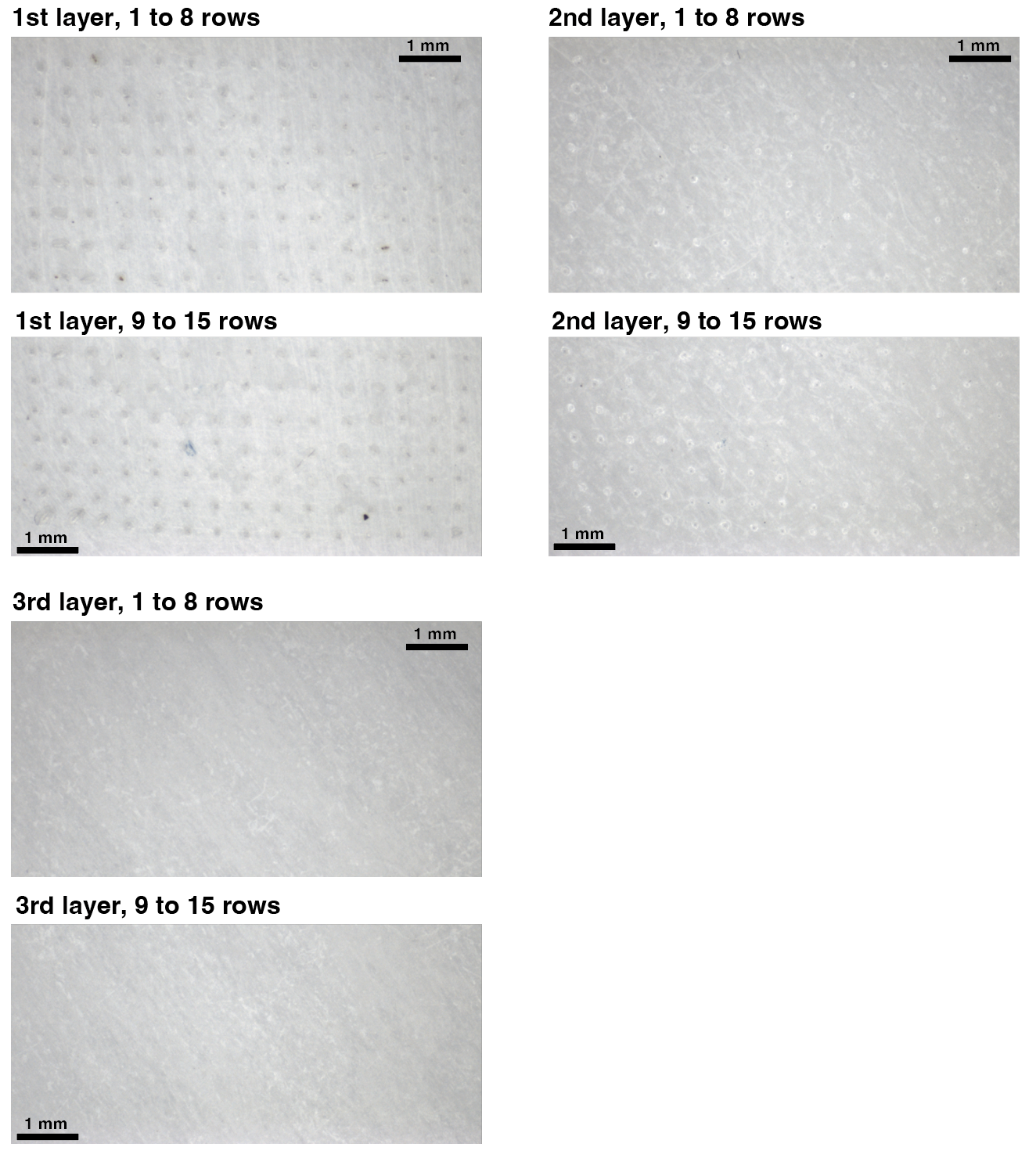


**Supplementary Figure 3.** Photograph of Parafilm® M layers after the insertion by the MN array of EEG type (1000 μm, 15 × 15 needles). Due to the field of view limitation of the optical microscope, the top 8 rows and the bottom 7 rows were imaged separately as two photographs.


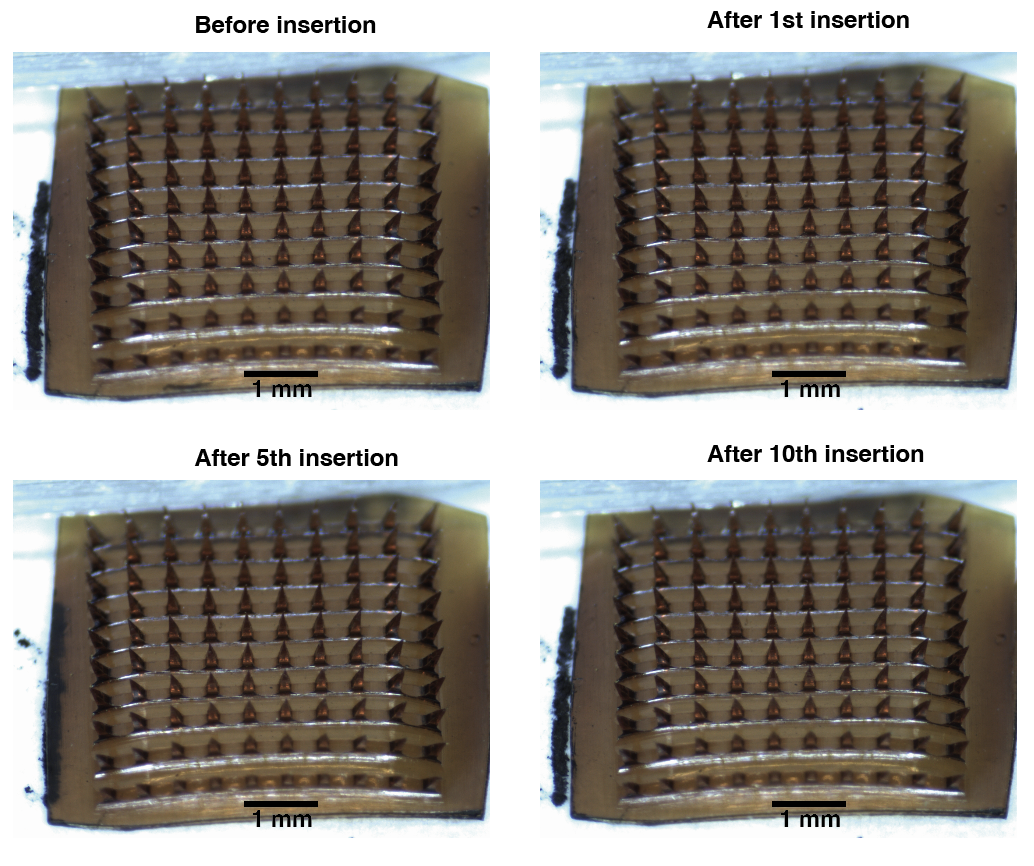


**Supplementary Figure 4.** Photographs of the polyimide MN array surface for EMG measurement after multiple applications to human skin.


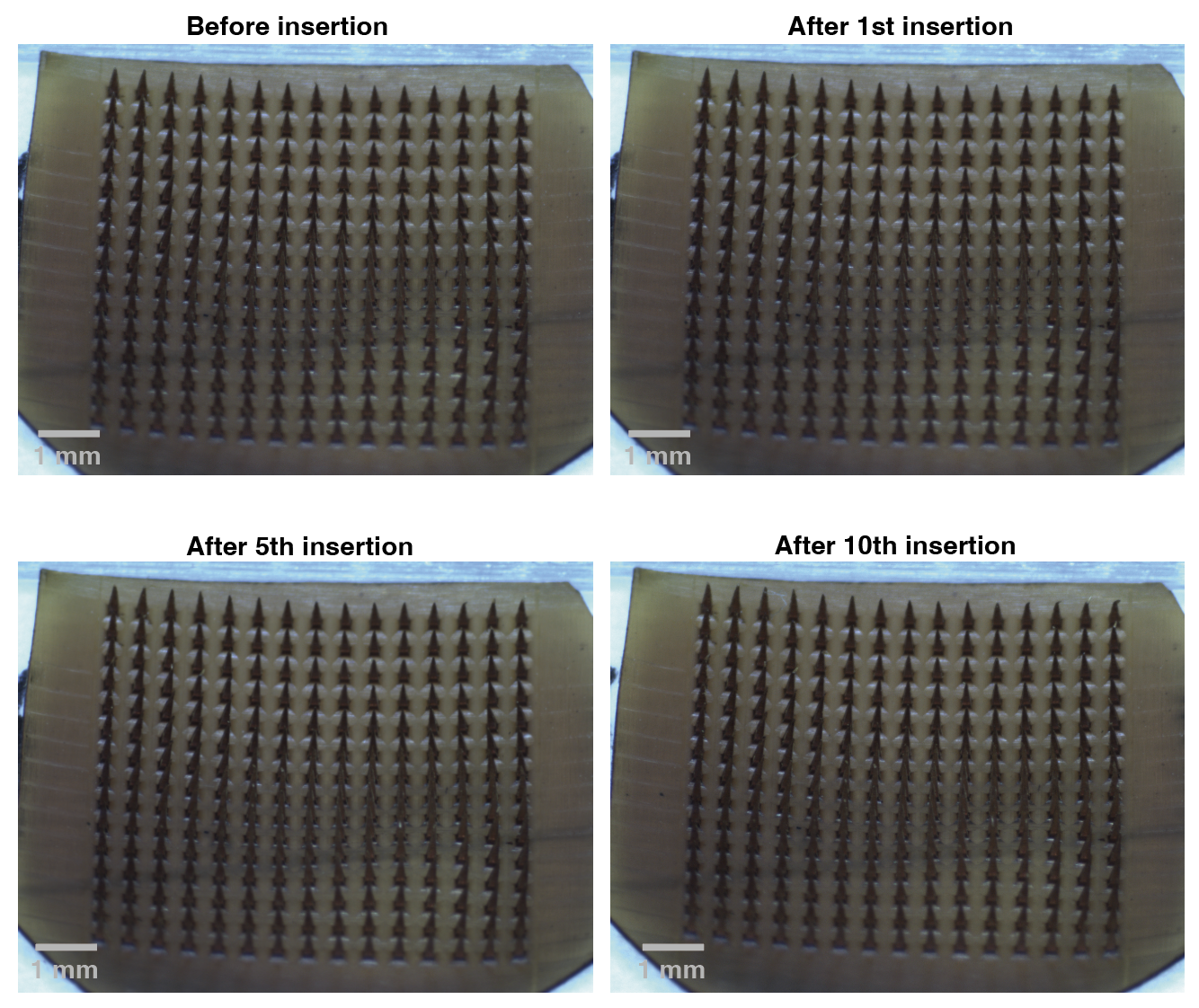


**Supplementary Figure 5.** Photographs of the polyimide MN array surface for EEG measurement after multiple applications to human skin.


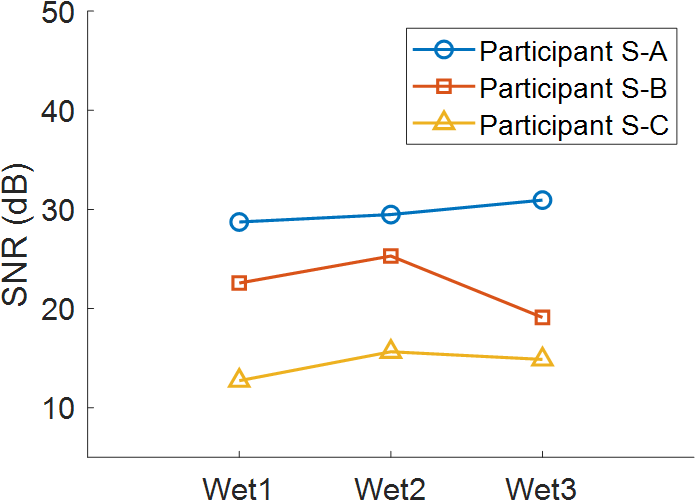


**Supplementary Figure 6.** Signal-to-noise ratio (SNR) of somatosensory evoked potentials (SEPs) of repeated SEP sessions.

All repeated sessions of SEPs ware measured with wet electrodes. Inter-session interval was 8 minutes. Each colored plot indicate individual data from three different participants.
